# Medical News

**Published:** 1851-05

**Authors:** 


### MEDICAL NEWS.

New Hospital.—It is generally understood that the additional hospital accommodation, which has been so long needed in Philadelphia, is about to be supplied under the auspices of the Protestant Episcopal Church—and upon the most liberal organization, "to be open to every creed, country, and color."

Dr. Elisha Bartlett has resigned the Professorship of the Institutes and Practice of Medicine in the New York University, which he has filled during the past year.

Dr. Thomas Reyburn has resigned the Professorship of Materia Medica and Therapeutics in the Medical department of the St. Louis University.

LIST OF COLLEGES, CLASSES AND GRADUATES FOR 1851.

| | Class. | Graduates. |
|---|---|---|
| Jefferson Medical College, . . . . . | 504 | 227 |
| University of Pennsylvania, . . . . . | 466 | 167 |
| University of New-York, . . . . . | 411 | 116 |
| N. York College of Physicians and Surgeons, . | 230 | 56 |
| New-York Medical College, . . . . . | 40 | 12 |
| Western Reserve College, . . . . . | 202 | |
| Starling Medical College, . . . . . | 125 | 35 |
| Castleton Medical College (2 sessions,) . . | 153 | 64 |
| Philadelphia College of Medicine (2 sessions,) . | 244 | 72 |
| Pennsylvania Medical College, . . . . | | 36 |
| Washington University of Baltimore, . . . | | 13 |
| Yale College, . . . . . . . | | 11 |
| Harvard University, . . . . . . | | 10 |
| Iowa University, . . . . . . . | | 10 |
| University of Virginia, . . . . . | 93 | 24 |
| Geneva Medical Institution, . . . . | 101 | |
| University of Buffalo, . . . . . | 115 | 30 |
| Albany Medical College, . . . . . | 93 | |
| Philadelphia College of Pharmacy, . . | 82 | 19 |
| Medical College of Georgia, . . . . . | 159 | 50 |
| Richmond Medical College, . . . . . | | 26 |
| Baltimore College of Dental Surgery, . . | | 17 |
| University of Maryland, . . . . . | | 45 |
| Ohio Medical College, . . . . . | 180 | 59 |
| University of Missouri, . . . . . | | 33 |
| Louisiana Medical College, . . . . . | | 37 |

*N. Y. Med. Gaz.*

Among the Original Communications in this number, will be found a reprint, from the American Journal of Medical Sciences, on the subject of establishing a lectureship on Dental Surgery in our Medical Schools, from the pen of Dr. Gardette, an eminent and highly educated practitioner in this department of Surgery in this city. We recommend it to the careful consideration of " those in authority."

An Act for the Registration of Births, Marriages, and Deaths, was passed by the Legislature of Pennsylvania, at the session just terminated.

The New Jersey Medical Reporter, one of our most acceptable exchanges, will hereafter be issued monthly instead of quarterly as heretofore.

Discouragement of Vicious Advertisements.—A Society has been formed in England, called "The Union for the Discouragement of Vicious Advertisements," which is designed to resist, and if possible do away with the disgusting parade of filthy advertisements with which the newspapers teem. This evil cries aloud for reform, no less in our own country than in England, and can be efficiently counteracted only by concentration and organization of effort. Will not some of the right-minded and public-spirited among us move in the matter?

A Pension of £100 per annum, on the Queen's Civil List, has been conferred on Mrs. Liston, the widow of the late Robert Liston, Esq., the eminent Surgeon.

Hospital for Sick Children, in London.—A number of distinguished noblemen and gentlemen in London, have taken in hand the organization of an hospital exclusively for sick children, "having the threefold object of affording relief to the poor, of promoting the advancement of medical knowledge, and of training up women to be efficient children's nurses." Such an institution is no less called for in Philadelphia, and we believe that proper effort is only wanting to commend so excellent an object to the action of the benevolent among us.

Dr. H. G. Clark, City Physician, of Boston, has been appointed one of the Surgeons of the Massachusetts General Hospital, in place of Dr. George Hayward, resigned.

Dr. Francis Gurney Smith has been appointed one of the Physicians of the St. Joseph's Hospital, Philadelphia, in place of Dr. S. Jackson resigned.

Use of Anæsthetic Agents in Ancient China.—Stanislas Julian has found, in examining the Chinese books in the National Library at Paris, the proof that the Chinese have been long acquainted with the use of anæsthetic agents during surgical operations. The extract which he gives is from a book published about the commencement of the sixteenth century, in fifty vols. quarto, and entitled "Kow-Kin-i-tong," "General account of Ancient and Modern Medicine," and refers to the practice of a celebrated physician, Ho-a-tho, who flourished between the years 220 and 230 of our era. It states, when about to perform certain painful operations, "he gave the patient a preparation of hemp" (hachich,) and that at the end of a few moments "he became as if he had been drunk or deprived of life." After a certain number of days the patient was cured, without having experienced the slightest pain during the operation.—*Edin. Phil. Journ.*

The Sale of Arsenic.—A bill to regulate the sale of arsenic has been introduced by the Ministry in the English Parliament, and will doubtless pass. It declares that the unrestricted sale of arsenic facilitates the commission of crime. The bill provides, that on every sale, particulars of the sale shall be entered by the seller in a book before the delivery of the arsenic, and every such entry is to be signed by the person selling the same. Any person selling arsenic, save as authorized in this bill, and every person giving false information to obtain arsenic, are to be summarily convicted before magistrates, and to be liable to a penalty not exceeding £20. The bill is not to prevent the sale of arsenic under a medical prescription. A recent case caused the introduction of the bill.

Medical Coroners.—"Judge Jackson stated emphatically in Court, on Thursday, during the progress of a trial in which reference was made to the Coroner's Court, that none but medical men ought to be appointed to the office of Coroner, as from their education they were peculiarly qualified to discharge efficiently the duties of the office. This opinion of his lordship appears to be acted on of late, very generally, both in Ireland and England, as medical men are elected almost everywhere that a vacancy occurs."—*Medical Times, from Limerick Chronicle*, March 8.

Obituary.—We are grieved to announce the decease of Professor J. B. Beck, of New York, on the 9th of April. The New York Academy of Medicine met on the 10th, and adopted resolutions expressive of their sense of this bereavement. His funeral was attended by the Academy in a body.

Our profession have recently lost another of its elder reputable members, by the decease of Dr. J. Smith Rodgers, of New York, in the 57th year of his age. He fell a victim to the ravages of carcinoma, by which he had been long a sufferer. He was long connected with many of the public institutions of New York.

We are pained to hear of the sudden demise of the venerable Dr. Jo-

SEPH BLOODGOOD, of Flushing, Long Island, where he had been so long an ardent and honorable member of the profession.

HENRY HOBART CURTIS, M. D., Deputy Health Officer at Staten Island, died on the 26th of April last, another victim to the ship fever, contracted in the discharge of his duty.

